# Child Motor Development before and after the COVID-19 Pandemic: Are There Social Inequalities?

**DOI:** 10.3390/children11080936

**Published:** 2024-07-31

**Authors:** Mariann Rigó, Simone Weyers

**Affiliations:** Institute of Medical Sociology, Centre for Health and Society, Medical Faculty and University Hospital Düsseldorf, Heinrich-Heine-University Düsseldorf, 40225 Düsseldorf, Germany; mariann.rigo@hhu.de

**Keywords:** child health, motor development, health inequalities, health equities, social disadvantage

## Abstract

Background: COVID-19 pandemic has influenced all children’s motor development. We examine whether this differs by social circumstances. We hypothesise that socially disadvantaged children experienced more pronounced motor problems compared with better-off children. Methods: Our trend study includes three representative waves of preschoolers (school years 2018/19, 2019/20, 2023/24) from a German school enrolment medical screening. Based on logistic regressions, we computed predicted prevalences of motor problems separately for children in different social circumstances (neighbourhood, migration background, family status). Results: Motor development of preschoolers slightly worsened by 2023 compared to pre-pandemic level (6.3 percent 2018 to 7.4 percent 2023). Results by neighbourhood highlight that preschoolers from well-off districts experienced a deterioration of motor development (4.9 percent 2018–6.4 percent 2023). The prevalence of motor problems is, however, below that of children from deprived neighbourhoods with consistently high prevalence (9.2–10 percent). Results by migration background show a worsening trend for children without migration background and the gap between the groups disappears in 2023. Results by family status do not indicate significant differences. Conclusion: Contrary to our hypothesis, socially disadvantaged children experienced smaller change in motor problems. However, our results highlight the potential of structured physical activities in daycare centres and sports facilities to enhance the motor development of these children.

## 1. Introduction

Physical activity in a structured way has been shown to be beneficial for children’s motor development [1]. The COVID-19 pandemic with its lockdowns limited the options for structured physical activity for young children; in the various waves, starting in March 2020, German daycare centres were closed for a couple of months. Later, they were operated in a restricted mode, e.g., they were only accessible for parents in key employments, or their activities were limited. The Corona Protection Ordinance expired in February 2023 [2]. Sports facilities were also closed or operated in a restricted mode for two years [3]. Similar infection control measures were introduced in other countries. As a consequence, a decline in all forms of physical activity among children was observed worldwide [4,5,6,7].

Correspondingly, a range of studies from different countries show that child motor development has deteriorated in the course of the pandemic. Evidence comes from Slovenia [8], Italy [9], Portugal [10], and Japan [11]. However, there are also studies showing little [12] or no effect of the COVID-19 pandemic [13]. These differences might be due to variations in lockdown policies between countries [12]. They might also be explained by the different quality of survey data and the measurement of the variables. 

While it has been postulated that children from vulnerable backgrounds will be most affected by the pandemic [14,15], there are only a few empirical studies applying an inequalities perspective and analysing motor development under different social circumstances. Comparing three smaller German samples, Wessely et al. [16] showed worse motor performance in cohorts under pandemic conditions, especially in children from socially deprived neighbourhoods. By contrast, comparing four Dutch cohorts over time, Uil et al. [12] found that children in the low socio-economic group of lockdown cohort 2 (2020–2021) showed increased motor skills development compared to the other cohorts. 

The evidence so far is mainly based on survey data with smaller samples. On the other hand, school enrolment medical screenings offer the advantage of having large samples, including children from different social circumstances [17]. Therefore, they are a valuable source of data in the context of child health inequalities.

In our previous research regarding pandemic health effects, we used routine school enrolment medical screening data from Dusseldorf and found that all groups of children experienced a deterioration in their motor development, and that there were no social differences between groups in the expected direction [18]. This study, however, was limited in terms of sample size and representativeness. In the pandemic years, the number of examinations was reduced, and they were mainly focused on vulnerable children. Therefore, our study on pandemic health effects only included vulnerable children, who were examined with priority. 

Since autumn 2022, school enrolment examinations in Dusseldorf have operated at full capacity, involving a complete group of preschool children. The availability of a representative wave after the pandemic facilitates the extension of our previous analysis. Furthermore, we can fill an important gap in the literature by adding a social-differential perspective to the analysis of children’s motor development throughout the pandemic. Based on our arguments above [14,15] and following the concept of differential vulnerability [19], we hypothesise that socially disadvantaged children experienced more pronounced deterioration in their motor development compared to better-off children.

## 2. Materials and Methods

Our study is based on observational cross-sectional data, which was collected during the school enrolment medical screening of children in the western German city of Dusseldorf. This examination is mandatory for all children before they enter school at around the age of six, and it is carried out by the municipal health authorities. Therefore, this is a data source representative of preschool children in Dusseldorf and is based on the objective assessment of a medical officer.

Six data waves of the school enrolment examinations are available. We executed our analysis using two waves before the pandemic (wave 1: enrolled at school in 2018/19, wave 2: enrolled at school in 2019/20) and one wave from the post-pandemic period (wave 6: enrolled at school in 2023/24). The samples under pandemic conditions in between (waves 3, 4, and 5 corresponding to school years 2020/2021, 2021/2022, and 2022/23) are smaller samples with a bias towards vulnerable children. Focusing on the three representative samples of preschool children allows us to have a more precise comparison of the different social groups. While our previous study [18] on vulnerable children was biased towards children in lower social groups and tended to only include the higher social groups and those with an indication for a health development problem, the availability of two pre-pandemic and one post-pandemic waves makes it possible to compare the full samples of children by socioeconomic circumstances. This facilitates a more precise estimation of the gap in their motor development. 

Motor development (the dependent variable) was assessed in the frame of the social paediatric screening, SOPESS [20]. The screening is designed to assess the overall development of children as part of the school enrolment medical screening in North Rhine-Westphalia. Characteristics of the screening include attention, knowledge of numbers and quantities, language, visuomotor skills and body coordination. The latter often serves to measure motor skills in school enrolment medical screenings. In SOPESS, body coordination is measured with one item in a standardised way by medical staff: the number of lateral jumps in a given time interval is counted. We used this one available item to operationalise motor development in our study. We applied the cut-off values given by the medical officer [21]: problematic motor development was indicated if the child scored less than 7 points out of 32. 

The school enrolment examination included three indicators of the child’s social circumstances (independent variables) that have been shown to be disadvantageous for child health (e.g., [22]): (i) *Neighbourhood* deprivation served as a proxy for family socio-economic position (SEP) [23]. It was assigned via the child’s home address and neighbourhood, based on indicators such as welfare benefits, living space per person, and migration population at the neighbourhood level. It ranges from 1 (no deprivation) to 5 (very high deprivation). We dichotomised the variable and compared 4/5 (high/very high deprivation) with 1-3 (no to medium deprivation); (ii) *Migration background* was operationalized using the survey item in their mother tongue. It is part of a basic set of indicators for children whose heritage is influenced by migration [24], and it is assessed by medical staff. We compared multilingual children with those speaking only German in their family; (iii) *Family status* was measured, contrasting single-parent families with two-parent families. This variable is also assessed by medical staff. 

As a first step of our analysis, to illustrate the difference in the composition and representativeness of the waves before, throughout, and after the pandemic, we computed the unadjusted prevalences of having problems with motor development and provided the sample distribution by social circumstances and by gender in each wave. 

To assess the trend in the motor problems of children, we estimated logistic regressions and calculated the adjusted predicted prevalence (APP) of children having problematic motor development. Our outcome variable is the dichotomous indicator of problematic motor development, and our independent variables are dummies for wave, gender, neighbourhood, migration background, and family status. To assess the differential trends by social circumstances, we interacted the wave dummy with the corresponding indicator of social group (neighbourhood deprivation, migration background, or family status). Following the recommendations by Mood [25], we report APP and average marginal effects (AME) instead of odds ratios (ORs). The use of APP and AME has several advantages compared to ORs. Besides being robust to unobserved heterogeneity, they are more intuitive, easier to interpret, and comparable across periods. Thus, we compute the APP of developing motor problems in the pre-pandemic and post-pandemic waves separately for the groups of children with disadvantageous social backgrounds (living in a deprived neighbourhood/having migration background/living in a single-parent family) and more advantageous social backgrounds (living in a well-off neighbourhood/not having migration background/living in a two-parent family). Then, based on the APP values, we compute the AME of a change in social circumstances in each wave, which is defined as the difference in the APP of motor problems between the more advantageous and the disadvantaged social groups. Thus, the AME of a change in social circumstances shows the gap in motor development between the social groups. Furthermore, we also compute the AME of a wave change within each social group. The AME of a wave change between wave 6 and wave 1 is the difference in the predicted probabilities of motor problems in wave 6 and in wave 1 for a specific social group. Our hypothesis envisaging that socially disadvantaged children experienced more pronounced deterioration in their motor development implies that the AME of a wave change (wave 6 vs. wave 1) is larger for the disadvantaged social groups compared to children living in more advantaged social circumstances. All analyses were conducted using Stata 18.

## 3. Results

### 3.1. Descriptives

Table 1 includes the descriptive statistics of all waves of the school enrolment examination. We observe that the pre-pandemic and post-pandemic full samples (2018/19, 2019/20, and 2023/24) include around 4500–4900 observations. However, the number of observations in the pandemic years (2020/21, 2021/22, 2022/23) is remarkably lower: between 1700 and 2900. The sample selection towards vulnerable groups is manifested by looking at the composition of the annual samples by social neighbourhood. While the number of examined children living in deprived neighbourhoods changed only slightly throughout the six waves (between 1144 and 1619), the number of examined children from well-off neighbourhoods dropped substantially in the pandemic years. Therefore, we decided to use the available representative samples for further analysis: the two pre-pandemic waves from 2018/19 and from 2019/20 and the post-pandemic wave of 2023/24. The raw descriptive statistics suggest that the prevalence of motor problems slightly increased from 2018/19 (6.2 percent) to 2023/24 (7.5 percent). 

**Figure 1:**
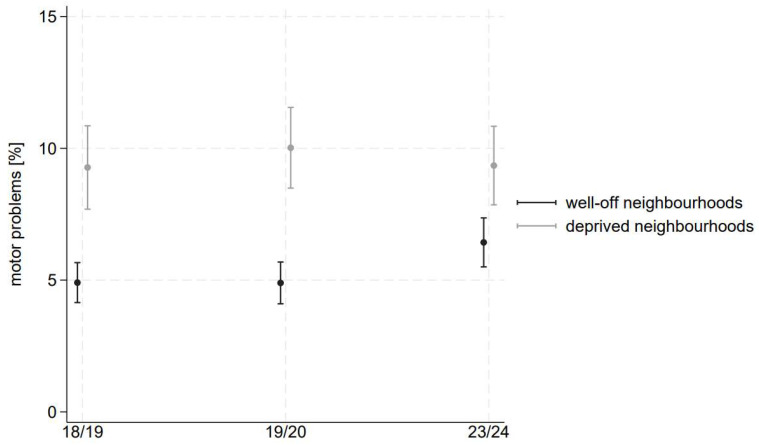
Adjusted predicted prevalence of motor problems by neighbourhood. Estimations based on logistic regression using the samples from 2018/19, 2019/20 and 2023/24. The estimations include an interaction between wave and neighbourhood, dummies for migration background, family status, and gender.

**Figure 2:**
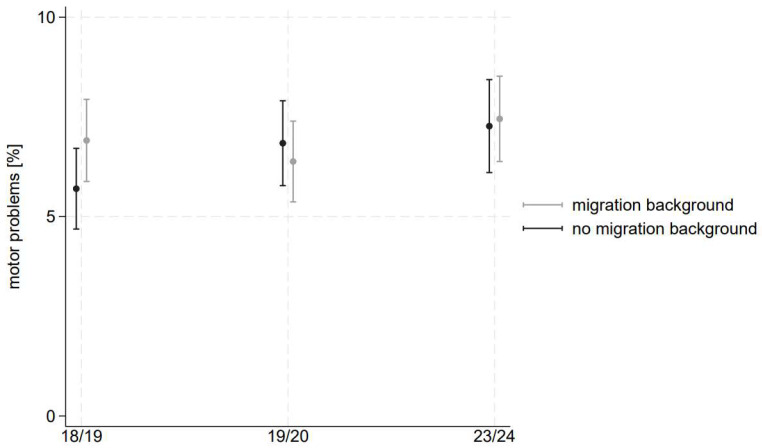
Adjusted predicted prevalence of motor problems by migration background. Estimations based on logistic regression using the samples from 2018/19, 2019/20, and 2023/24. The estimations include an interaction between wave and migration background, dummies for family status, neighbourhood, and gender.

**Figure 3:**
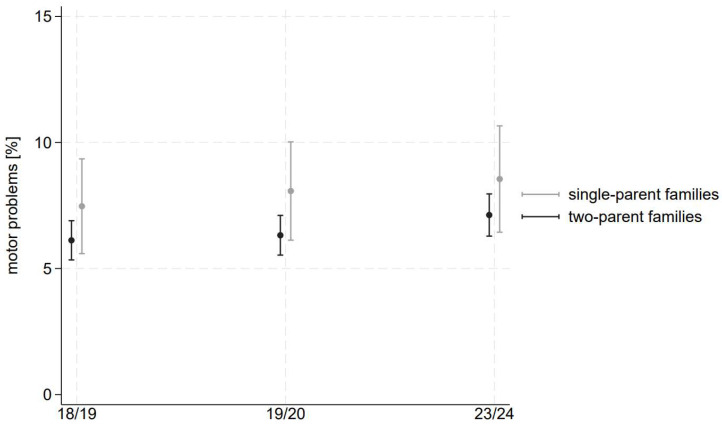
Adjusted predicted prevalence of motor problems by family status. Estimations based on logistic regression using the samples from 2018/19, 2019/20, and 2023/24. The estimations include an interaction between wave and family status, dummies for migration background, neighbourhood, and gender.

The results of our logistic regressions yield further insight into the differential trends based on social circumstances. We show the results in Figure 1, Figure 2 and Figure 3 as well as in Table 2, including the AMEs.

### 3.2. Neighbourhood

As illustrated by Figure 1, we observe a significantly higher prevalence of motor problems among children living in deprived neighbourhoods compared to children living in well-off neighbourhoods. As indicated by the AME of living in a deprived vs. well-off neighbourhood (see Table 2), the gap between the two groups of children is decreasing over time. While in the pre-pandemic wave 2018/19 (2019/20), the prevalence of motor problems was 4.4 (5.1) percentage points higher among children living in disadvantaged districts, the AME (deprived vs. well-of neighbourhood) decreased to 2.9 percentage points by the post-pandemic wave. The gap (AME) is, however, still significant in the last wave, pointing to a consistently higher prevalence of motor problems among children from disadvantaged districts. 

Examining the development of motor problems over time, we observe a deteriorating trend among children living in well-off districts. The comparison of the last and first waves indicates that the prevalence of motor problems increased from 4.9 percent to 6.4 percent, with a 1.5 percentage point deterioration. Regarding children living in socially deprived neighbourhoods, the predicted prevalence of motor problems remains consistently high at around 9.2–10 percent in all three waves. 

### 3.3. Migration Background

We observe a small gap between children with and without migration backgrounds in the first wave, indicating a 1.2 percentage point higher prevalence of motor problems among children with migration background (see Figure 2 and Table 2). This gap disappears in consecutive waves. 

The time development of motor problems points out a worsening trend for children without migration background. They experience a 1.6 percentage point higher prevalence in motor problems in wave 6 (2023/24) compared to wave 1 (2018/19). Among children with migration backgrounds, we did not detect significant changes in their motor development between the waves. 

### 3.4. Family Status

We do not observe significant differences between the two groups of children living in single-parent or two-parent families (see Figure 3 and Table 2). Though the prevalence of motor problems tends to be somewhat higher among children in single-parent families, the difference (as indicated by the AME of living in single-parent vs. two-parent families) is statistically not significant. This might be due to the relatively small number of single-parent families in our sample and the even smaller number of motor problems among them, which can lead to wide confidence intervals and, thereby, to insignificant estimates. Considering the trends throughout the waves, a slight deterioration can be observed in both groups. 

We summarised all results including the AMEs in Table 2 below. 

### 3.5. Robustness Check

We have carried out robustness checks to see if the general trend of motor problems differs by gender. We have found significant differences in the motor development of boys and girls. However, the magnitude of the gap was relatively stable through all three representative waves. In general, the prevalence of motor problems was about 1.8–2.5 percentage points higher among males compared to females. Therefore, the AMEs (between waves, between social groups) are not affected by gender differences. Consequently, our main findings have not changed due to gender differences. The gender-specific results are available from the authors upon request. 

## 4. Discussion

The aim of the study was to examine the changes in children’s motor development by social circumstances throughout the pandemic. We hypothesised that socially disadvantaged children experienced more pronounced deterioration in their motor development compared to those with better social circumstances.

### 4.1. Key Results

Our results indicate that the motor development of preschool children in Dusseldorf, on average, deteriorated during the pandemic. Contrary to our hypothesis, however, our results indicate that the gap in motor development between children with different social circumstances decreased or was insignificant throughout the pandemic. Results stratified by neighbourhood highlight that preschoolers living in well-off neighbourhoods experienced a significant deterioration in their motor development. However, their prevalence of motor problems is much lower than that of children living in deprived neighbourhoods. These children show consistently high prevalences across all waves. Results by migration background also point to a slight worsening for children without a migration background, and the gap between the two groups disappears after the pandemic. Results by family status do not indicate significant differences regarding the level and trend of motor problems between one- and two-parent families. 

### 4.2. Strengths and Limitations

An important strength of the study is that it is based on actual administrative data with complete samples, including children from all social groups. This provides a unique opportunity to analyse inequalities in motor development in a timely and efficient manner using representative samples. However, a limitation of using these administrative data is that they were not representative during the pandemic years. Therefore, we had to limit our analysis to two waves before and one wave after the pandemic period.

Another strength of our study is that we have an objective measure of motor development, which is the result of a standard medical procedure. However, the available measure of body coordination is limited. In general, motor development includes a range of coordinative and conditional skills. This range is, however, not assessed in the frame of the school enrolment examinations, where only one single measure of motor skills is available. We believe that the advantage of having a representative sample outweighs this limitation. As a consequence, we were not able to differentiate between aspects of motor development as similar studies have (e.g., [10,11]).

In addition, three different indicators of social circumstances could be included in the analyses to show their different relationships with motor development. However, two indicators are methodologically limited: (i) Neighbourhood deprivation is only a proxy for the socioeconomic position of the family, with the risk of an ecological fallacy. Due to data protection, individual-level SEP data were not available; (ii) With regard to migration background, we could only use family language as one basic indicator [24]. Other indicators, such as parents’ country of birth, were not available. However, following Schenk [24], we argue that migration brings with it specific life conditions that impact health and language reflects these conditions beyond parents’ birth country. 

Furthermore, the number of available confounders is limited; apart from the few variables describing the children’s social circumstances, the school medical examinations do not assess detailed information on their family situation (e.g., living environment) or health behaviour (e.g., physical activity). Also, we do not know whether the parents worked in system-relevant occupations and whether their children were entitled to use daycare facilities during the pandemic. Therefore, we could not adjust our regressions for these variables. 

### 4.3. Interpretation

Generally, our results highlight that the motor development of preschool children in Dusseldorf deteriorated during the pandemic. Our post-pandemic sample includes children who were enrolled in first grade in the school year 2023/24. This means that during the pandemic, these children were 3 to 4 years old. This age group has proven to be particularly vulnerable in terms of developmental problems during the pandemic. Studies using developmental diagnostic tests [26] or medical assessment [27] showed that the motor development of toddlers has deteriorated more than that of infants. In what follows, we will systematically discuss the inequality implications of our results.

#### 4.3.1. Neighbourhood

We expected a stronger increase in motor problems in socio-economically disadvantaged children. Neighbourhood and home resources for physical activity are worse for socio-economically disadvantaged children than for better-off children. Even before the pandemic, there was evidence that socio-economically disadvantaged children lacked facilities such as gardens [28] and green spaces [29] at home. In the course of the pandemic, a German study showed that children from socially disadvantaged families performed worse in terms of their physical activity, especially if they did not have access to their own garden or lived in large apartment complexes [30]. Similarly, a Canadian study [31] showed that, for children, houses (versus apartments) were correlated with increased outdoor activities, while proximity to major roads was a barrier. Also, parents with lower incomes were more hesitant to return their children to active play during the pandemic [32]. It was therefore not surprising that, e.g., Wessely et al. [16] found a steeper decrease in lateral jumping performance in children from deprived neighbourhoods. 

What we found in our data, however, was a deteriorating trend among children living in *well-off neighbourhoods*. This effect is counterintuitive. To explain it, we should first consider what has changed for children during the pandemic. While the home environment remained the same, daycare centres and recreational sports facilities were closed. The consequence of these closures can vary by the children’s socio-economic position. 

Sports facilities: Previous evidence pointed out that, in general, socio-economically disadvantaged children use organised recreational sport less often than better-off children [33]. Therefore, the lockdown has brought about significantly less changes for disadvantaged children than for better-off children. In the lives of better-off children, sports activities, which used to be an important resource for promoting motor development, have ceased for a long while. 

Daycare centres: Two explanations are possible here. First, children from socio-economically disadvantaged families are generally underrepresented in daycare centres [34]. Accordingly, less socio-economically disadvantaged preschoolers have probably been affected by the daycare centre closures compared to better-off ones. Second, depending on the neighbourhood-level SEP, the quality of care in the facilities differs, and children from less privileged families receive less stimulation (including motor development) in the facilities they attend [35]. Although a survey of educational professionals showed that the promotion of linguistic, socio-emotional, and motor development has taken the highest priority in everyday daycare, even during the pandemic [36], it could be argued that this has varied depending on the facility and clientele. Thus, the above-mentioned differences in daycare quality under normal conditions have probably also played a role during the pandemic.

It can be concluded that the lack of sport, quality childcare, and other resources for socio-economically disadvantaged children, even under normal circumstances, is so severe that they experience a level of motor problems that better-off children face under pandemic conditions. Conversely, our study results for children in well-off neighbourhoods demonstrate what happens when services are eliminated.

#### 4.3.2. Migration Background

Results regarding migration background are again counterintuitive. Families with a migration background live in more cramped conditions than families without a migration background. Their living space per person is significantly less than that of natives, they tend to live in apartment blocks, and they are less likely to have a garden [37]. Therefore, we expected to observe a sharper increase in motor problems in the case of migrant children when they were thrown back to their home environment during the pandemic. Also, similar to children with low SEP, migrant children use organised recreational sports less often than children without a migration background [33]. They are also underrepresented in daycare centres compared to children without a migration background. More than two-thirds of parents do not take up the option of childcare in a daycare centre because of barriers to accessing a place [38]. Thus, similar to socio-economically disadvantaged children, the lockdown has brought about fewer changes for children with migration backgrounds. On the other hand, the situation has changed substantially for German children, who are overrepresented in organised recreational sports and high-quality daycare centres under normal conditions. Again, results point to the importance of organised recreational sports and access to (high-quality) daycare.

#### 4.3.3. Family Status

Finally, our results show that children from single-parent families are especially challenged at any time. Even before the pandemic, we had seen the developmental disadvantages of children growing up in single-parent families. Emotional or behavioural problems and chronic diseases are more prevalent [39], and poor health status and obesity are more frequent [40]. 

In the course of the pandemic, studies interviewing single parents with preschool-aged children have shown that they struggle to provide constant childcare, especially with regard to organising meaningful activities. Subsequently, screen time is extended, which provides parents with time to concentrate on paid work and household duties. Children were reported to be left alone without care for longer periods of time [41]. Studies specifically comparing single- and dual-parent households show that single-parents were more hesitant to return their children to active play and organised sports during and after the pandemic [32,42].

Bearing this in mind, our results, suggesting a stable gap in coordination problems between single-parent and two-parent families over time, are surprising. They can be interpreted in light of our previous argument: If extra-familial activities, such as leisure sports and daycare centres, are eliminated, children are affected, regardless of whether they grow up in single- or two-parent families.

## 5. Conclusions

First, our results point out that the age group of toddlers should receive special attention in terms of motor development promotion. The results along with neighbourhood and migration background highlight the importance of recreational sports in general. This is in line with previous evidence indicating that structured physical activity enhances children’s gross motor skills longitudinally [1]. Our results show that the temporary shortage of structured physical activity leads to more frequent motor problems among those children who are more likely to utilise these facilities under normal conditions. However, more importantly, our results draw attention to the unfavourable situation of children living in disadvantageous circumstances. These children, even in normal times, are severely underrepresented in recreational sports facilities and face motor problems even more often than children in better social circumstances under the pandemic. Therefore, increasing the participation of socio-economically disadvantaged children could lead to a reduction in motor development problems in general. However, this requires affordable offerings in recreational sports. Studies show that sports facilities are less affordable for families at risk of poverty [43]. 

Second, the results underline the need to reduce structural barriers to daycare attendance for socially disadvantaged and migrant families and to develop quality standards for promoting physical activity within daycare centres [44]. Results along family status emphasise the importance of creating adequate supports for parents from various social circumstances to ensure that children in those families can take advantage of the health benefits of physical activity [42]. 

## Figures and Tables

**Table 1:** Raw descriptive statistics of the waves from school year 2018/19 to 2023/24.

	Wave 1 2018/19		Wave 2 2019/20		Wave 3 2020/21		Wave 4 2021/22		Wave 5 2022/23		Wave 6 2023/24	
	No.	%	No.	%	No.	%	No.	%	No.	%	No.	%
**Motor problems**												
no	4216	93.8	4130	93.4	2342	90.6	1323	89.6	2432	92.5	3983	92.5
yes	279	6.2	294	6.6	242	9.4	153	10.4	197	7.5	322	7.5
total	4495	100	4424	100	2584	100	1476	100	2629	100	4305	100
**Neighbourhood deprivation**												
well off	3444	70.9	2980	66	1233	45.3	584	33.8	1338	45.9	2876	64
deprived	1413	29.1	1538	34	1490	54.7	1144	66.2	1574	54.1	1619	36
total	4857	100	4518	100	2723	100	1728	100	2912	100	4495	100
**Migration background**												
no	2605	53.3	2358	51.7	1275	46.1	804	45.8	1469	50.0	2191	48.1
yes	2286	46.7	2206	48.3	1492	53.9	950	54.2	1471	50.0	2360	51.9
Total	4891	100	4564	100	2767	100	1754	100	2940	100	4551	100
**Family status**												
two-parent	4153	84.9	3843	84.2	2338	84.5	1428	81.4	2466	83.9	3866	84.9
single-parent	738	15.1	721	15.8	429	15.5	326	18.6	474	16.1	685	15.1
total	4891	100	4564	100	2767	100	1754	100	2940	100	4551	100
**Gender**												
male	2583	52.8	2345	51.4	1437	52	1004	57.3	1557	53.6	2344	52.1
female	2307	47.2	2217	48.6	1329	48	748	42.7	1349	46.4	2157	47.9
total	4890	100	4562	100	2766	100	1752	100	2906	100	4501	100

**Table 2:** Adjusted predicted prevalences of motor problems by SEP.

Motor Problems [%]					
	Wave 1 2018/19	Wave 2 2019/20	Wave 6 2023/24	AME W6 vs. W1 (*p*-Value)	AME W6 vs. W2 (*p*-Value)
**Neighbourhood deprivation**					
well-off neighbourhoods (se)	4.905	4.893	6.430	1.525	1.537
	(0.387)	(0.404)	(0.474)	(0.012)	(0.013)
deprived neighbourhoods (se)	9.273	10.021	9.346	0.072	−0.675
	(0.807)	(0.781)	(0.761)	(0.947)	(0.531)
AME deprived vs. well off (*p*-value)	4.369	5.128	2.915		
	(0.000)	(0.000)	(0.001)		
**Migration background**					
no	5.700	6.841	7.269	1.570	0.429
(se)	(0.516)	(0.543)	(0.595)	(0.044)	(0.593)
yes	6.909	6.381	7.451	0.542	1.070
(se)	(0.525)	(0.517)	(0.546)	(0.471)	(0.151)
AME yes vs. no (*p*-value)	1.210	−0.459	0.182		
	(0.103)	(0.542)	(0.823)		
**Family status**					
two-parent families	6.119	6.320	7.123	1.004	0.803
(se)	(0.397)	(0.402)	(0.427)	(0.085)	(0.171)
single-parent families	7.470	8.075	8.550	1.080	0.475
(se)	(0.959)	(0.995)	(1.076)	(0.453)	(0.745)
AME single vs. two parents (*p*-value)	1.351	1.755	1.427		
	(0.193)	(0.102)	(0.218)		
N (observations)	13,049				

Notes: Estimations based on logistic regression using the samples from 2018/19, 2019/20, and 2023/24. The estimations include an interaction between wave and the corresponding indicator of SEP, dummies for SEP (not included in the interaction) and gender. Adjusted predicted prevalences are reported with standard error in brackets (se), AMEs are reported with *p*-value in brackets.

## Data Availability

The original contributions presented in the study are included in the article, further inquiries can be directed to the corresponding author/s.
